# A one health roadmap towards understanding and mitigating emerging Fungal Antimicrobial Resistance: fAMR

**DOI:** 10.1038/s44259-024-00055-2

**Published:** 2024-11-07

**Authors:** Matthew C. Fisher, Fiona Burnett, Clare Chandler, Neil A. R. Gow, Sarah Gurr, Alwyn Hart, Alison Holmes, Robin C. May, Janet Quinn, Tarek Soliman, Nicholas J. Talbot, Helen M. West, Jon S. West, P. Lewis White, Michael Bromley, Darius Armstrong-James

**Affiliations:** 1 MRC Centre for Global Infectious Disease Outbreak Analysis, Imperial, London, UK; 2 Scotland’s Rural College (SRUC), West Mains Road, Edinburgh, UK; 3 Department of Global Health & Development, London School of Hygiene & Tropical Medicine, 15-17 Tavistock Place, London, UK; 4 Medical Research Council Centre for Medical Mycology, University of Exeter, Geoffrey Pope Building, Stocker Road, Exeter, UK; 5 Biosciences, University of Exeter, Exeter, UK; 6 Chief Scientist’s Group, Environment Agency, Bristol, UK; 7 Pharmacology and Therapeutics, University of Liverpool, Liverpool, UK; 8 School of Biosciences, The University of Birmingham, Birmingham, UK; 9 Biosciences Institute, Faculty of Medical Sciences, Newcastle University, Newcastle upon Tyne, UK; 10 The Sainsbury Laboratory, University of East Anglia, Norwich Research Park, Colney Lane Norwich, UK; 11 School of Biosciences, University of Nottingham, Sutton Bonington Campus, Loughborough, UK; 12 Protecting Crops and the Environment, Rothamsted Research, Harpenden, UK; 13 Public Health Wales Mycology Reference Laboratory, University Hospital of Wales, Cardiff, UK; 14 Manchester Fungal Infection Group, University of Manchester, Manchester, UK; 15 Department of Infectious Diseases, Imperial, London, UK

**Keywords:** Antimicrobial resistance, Fungal infection

## Abstract

The emergence of fungal antimicrobial resistance—fAMR—is having a growing impact on human and animal health, and food security. This roadmap charts inter-related actions that will enhance our ability to mitigate the risk of fAMR. As humanity’s reliance on antifungal chemicals escalates, our understanding of their one-health consequences needs to scale accordingly if we are to protect our ability to manage the global spectrum of fungal disease sustainably.

## Background

The Kingdom Fungi is a biodiverse and essential component of our habitable planet. However, recent decades have seen an increase in the number of pathogenic fungi impacting natural populations and managed landscapes: fungi are increasingly recognized as presenting a worldwide threat to food security as well as the healthy functioning of ecosystems^1,2^. In parallel, clinicians and healthcare scientists are struggling to treat emerging and established fungal pathogens, that kill millions of people every year^3^ and are becoming increasingly adapted to resist frontline antifungal therapies^4–6^. The causes that underpin this surge of fungal disease are diverse, complex and multisectoral. However, an overarching explanation for the changing epidemiology of mycoses is provided by the sheer size of the fungal kingdom—fungi total around 20% of eukaryotic life on earth, numbering between 2 and 3 million species, of which less than 5–10% have been taxonomically identified^7^. That fungal associations underpin nearly all life on Earth speaks to this kingdom’s existence for over a billion years and its extraordinary evolutionary plasticity: this adaptability largely owes to the capacity of fungi to synthesise and then secrete a broad spectrum of biochemical molecules with which they manipulate, feed on, and supply to other domains of life, thus playing an essential role in biodegradative nutrient recycling. By combining complex biochemical traits with evolutionarily labile genomes and diverse morphologies, ranging across yeasts, mycelial networks, mushrooms and hardy aerosolised spores, this mélange of adaptive traits has created a formidable biological force on earth.

In order to manage our intimate association with fungi, humans are dependent on chemical methods of control. Pre-modern defenses used readily available substances such as urine, salts (brining), honey, lactic acid bacteria and nitrites to preserve stored foods from moulds, then copper and sulphur compounds to defend crops^8^. The first antifungals used in humans, the polyenes, were discovered in the 1950s with subsequent pharmaceutical advances leading to the four main classes of licensed clinical antifungals in use today for the treatment of life threatening infections (Table 1). Parallel development has led to the innovation of many diverse fungicidal chemicals with 10 known broad modes of action (MOA) that are used across agriculture^9^. Of these, three primary classes of fungicide predominate in agriculture, comprising over 70% of the global market for fungicides: the demethylation inhibitors (DMIs) that target sterol biogenesis, and two classes of respiratory inhibitors: the “quinone outside” inhibitors (QoIs) and succinate dehydrogenase inhibitors (SDHIs). The worldwide use of antifungal chemicals in the environment combined with the adaptability of fungal genomes has led to the emergence of fungal antimicrobial resistance (fAMR) as a rapidly evolving one-health threat, spanning food security, the built and natural environments, human and animal health^10^. Fungal pathogens exhibit exceptional adaptability to environmental stressors and have shown a remarkable capacity to overcome existing and novel antifungal drugs through diverse mechanisms^11^. Owing to the capability of fungi to disperse as resilient spores, or through direct contact and by hijacking anthropogenic trade routes, the spread of novel adapted traits which include fAMR now occurs worldwide and is challenging to control^12^. It is increasingly clear that we face a complex global problem where protecting ourselves and our foods against fungal pathogens results in strong selective pressures, leading to an erosion of our antifungal protection by evolving fAMR.

**Table 1:** Main modes-of-action (MOAs) of antifungal chemicals in the clinic and the environment, and where dual-use may occur

MOAs (FRAC code)	Class	Examples		Dual use
		Clinical	Agricultural	
Demethylation Inhibitors (DMIs) of fungal membrane sterol biosynthesis (G)	Azoles	Imidazole	Imazalil Prochloraz	Yes
	Triazoles	Fluconazole Itraconazole Voriconazole Posaconazole Isavuconazole	Tebuconazole Tetraconazole	Yes
		Opelconazole (*in development*)	Piperazines	Possible
		-	Pyridenes	No
		-	Pyrimidines	No
		- -	Triazolinthiones	No
	Allylamine	Terbinafine		
Cell wall biosynthesis (H)	Echinocandins	Caspofungin Micafungin Anidulafungin Rezafungin	-	No
	Carboxylic acid amides	-	Mandipropamid	No
	Triterpene	Ibrexafungerp		
Cell membrane disruption (F)	Polyenes	Amphotericin Nystatin	-	No
	Phosphorothiolates	-	Iprobenfos	No
Nucleic Acids metabolism (A)	Pyrimidine analogues/Phenylamides	Flucytosine Tavaborole	Metalaxyl	Possible
	DHODH inhibition	Olorofim (*in development*)	Ipflufenoquin Quinofumelin	Possible
Cytoskeleton and motor proteins (B)	MBC fungicides	-	Benomyl	No
Respiration (C)	Succinate dehydrogenase inhibitors	-	Fluxapyroxad Boscalid	No
	Quinone outside inhibitors	-	Azoxystrobin	No
Protein transfer	GWT-1 inhibition	Fosmanogepix (*in development*)	Aminopyrifen (*in development*)	Possible

The list of agricultural fungicide MOAs is limited to the main widely-used categories, with the full list accessible at^9^.

In this Perspective, our aim is to integrate the views of a panel of scientists from the United Kingdom (UK) with broad expertise in mycology, agriculture, public health/healthcare and social science in order to outline a roadmap for addressing fAMR within the ‘one health’ paradigm. We use this roadmap to highlight where opportunities lie to operationalise this knowledge to understand benefits and trade-offs, with the combined aims of securing food production and reducing ecological impacts whilst saving animal and human lives. While our focus is on the UK, our insights are of broad relevance to the global setting and ongoing international activity to better understand emerging fAMR.

## fAMR in the environment

Plant-pathogenic fungi account for around 20% crop yield destruction, with an additional 10% loss post-harvest, signifying a major threat to food security^13^. Modern crops are characterised by high genetic uniformity (monocultures) and the key global staples, cereals and root-crops, are predominantly monocultures planted at vast scales. Consequentially, there is the opportunity for rapid expansion of those phytopathogens with fast reproduction cycles and plastic genomes, leading to the selection of fungicide-resistant and highly-virulent strains^14,15^. Here, the deployment of disease resistance encoded by single dominant genes, coupled with fungicides with single modes of action, provides the ideal scenario for such selection to occur.

The emergence of antifungal resistance to azole DMI fungicides in important wheat fungal pathogens such as *Zymoseptoria tritici* is well recognised. Annual EU yield loss associated with *Z. tritici* blotch is currently €1.6 billion owing to extensive resistance to the most common group of DMIs, the azoles^15^. The global value of wheat fungicides targeting *Z. tritici* is 1.2 billion US dollars; UK wheat, for example, received 1,968,827 kg fungicides in 2022. Following the introduction of the DMI imazalil in the 1970s, there are currently more than 25 different azoles holding a combined market share of around 25%, but manifesting year-on-year increases in global sales. These increases have been marked in certain regions, with a 400% rise to ~3000 metric tons per year from 2006 to 2016 seen in North America^16^ and an estimated 30,000 metric tons used per year in China^17^, with comparable trends repeated in the European Union^18^. The introduction of the strobilurin group of QoI fungicides in the late 1990’s, which showed exceptional broad spectrum activity against a wide range of fungal pathogens of major crops, was followed by the emergence of fAMR less than two years after release. Strobilurin resistance is now widespread because it arises so readily by a single mutation in the cytochrome b gene; for instance in the wheat blast pathogen *Magnaportha oryzae* that now threatens wheat production across three continents^19^. While securing our harvests is underpinned by successful control of fungal pathogens, agriculture is locked into cycles where escalating levels of fAMR require increasing dependence on chemical fungicides alongside the continual development of novel resistant crop cultivars. Overall, it is clear that current products sold by agribusiness and protocols adopted by modern agricultural practice are failing in their aim of providing evolutionarily resilient methods of securing our future harvests.

The dependence on fungicides that target single molecular targets (‘single-site fungicides’) in modern agriculture is mirrored in other sectors^20^. For instance, azoles are strong ligands for copper ions and are combined to produce potent antifouling agents for watercraft to prevent biofilm accumulation^21^; in the home, antifungal chemicals are included in ‘anti-mould’ paints that can be bought from most DIY shops; in the timber industry, wood preservatives are a mix of copper and azoles^20^; in horticulture, plant bulbs are routinely dipped or sprayed with azole fungicides as protectants^22^. While the persistence of fungicides is highly variable in the environment and depends on a multitude of factors including the soil-type and its microbiome, DMIs are robust cyclic molecules. The modern fluorinated forms such as mefentrifluconazole have a degradation half-life (DT_50_) range of 104 – 477 days, a DT_90_ >1000 days and are persistent in soils^23^. Given: (i) the ubiquity of fungicide use in a wide range of environments and human products, (ii) the quantities involved and (iii) the kinetics of their degradation, it is not surprising that increasingly these chemicals are near systemic micropollutants of soils, recycled green-waste, homes and waterways where they will exert strong directional natural selection on the fungal kingdom—whether targeted by the original fungicide application or not. This pan-kingdom exposure to broad-spectrum fungicides raises many questions as to their ecotoxicological risk to fungal biodiversity per se. For this reason, it is clearly important to better understand the extent of antifungal exposure in the environment and its contribution to not only antifungal resistance in targeted plant-fungal pathogens, but to those off-target fungi that occur ubiquitously in the natural environment and are not only essential for soil ecosystem services but may also cause serious disease in humans^5^.

## fAMR in human mycoses

In 2022, the World Health Organization (WHO) published the first fungal priority pathogens list (FPPL) with the aim of focusing research and policy interventions to strengthen the global response to fungal disease^24^. Recent estimates of the global burden of deaths that are directly attributable to fungal disease are in the region of 2.5 million, with over 6·5 million people affected each year by life-threatening fungal diseases leading to an estimated 8–49 M disability-adjusted life years (DALYs)^3,25^. Human pathogenic fungi adapt readily to chemical pressure and fungal antimicrobial resistance has been shown to develop to all forms of clinical antifungals currently in usage^10^. Of the 19 listed pathogens, the WHO critical group includes the yeasts *Cryptococcus neoformans*, *Candida auris*, *C. albicans* and the filamentous mould *Aspergillus fumigatus*, all of which are known to readily adapt to antifungal drug pressure, and for which fAMR was of high public health concern owing to its link to increased morbidity and mortality^4^. There is consensus that fAMR in *A. fumigatus* and *C. auris* is an emerging global public health crisis that require immediate action; the World Health Organisation (WHO) has highlighted these species as a particular concern in its ‘global research agenda for AMR in human health’^26^ alongside the listing of both of these pathogens on the urgent AMR threat list published by the US CDC in 2019^27^.

Fungal infections, both superficial and invasive, are known to evolve durable resistance to antifungal therapies following long-term therapy. This may be the consequence of antifungal treatments being given as monotherapies alongside a high frequency of subtherapeutic levels of antifungal drugs, which has been linked to a higher risk of developing a resistant infection^28,29^. Infections by many fungi—yeasts, dermatophyte skin mycoses and some respiratory pathogens (e.g., *Pneumocystis jirovecii*)—are transmitted person-to-person and can quickly spread. For instance, global expansion of *C. auris* post its first detection in 2009, is paralleled by an increase in resistance to first-line echinocandin antifungals^30^. The nosocomial spread of these drug-resistant *C. auris* variants is enhanced by rampant dissemination in healthcare settings where mechanical ventilation is used, and where there is contamination from surfaces and biofilms of the organism^31,32^. A further recent example of novel emerging fAMR is the terbinafine-resistant *Trichophyton indotineae* dermatophyte that is rapidly spreading in humans across multiple continents^33^. The rapid emergence of this inflammatory dermatophyte has been linked to the overuse of topical creams containing combinations of antifungals, antibacterials, and corticosteroids^34^.

Emerging clinical fAMR has been linked to changing exposures to fungi in the environment. Exposure to mould in homes has emerged as an important public health issue, reflecting worsening social inequality (e.g. poor housing in deprived areas) alongside climate-dependent processes that lead to increased damp, humidity and flooding^35^. It is recognised that better regulatory requirements are needed to mitigate household mould exposures as these have clinical and domestic impacts that include precipitation of first episode and worsening asthma, environmental chemical hypersensitivity syndrome, and “sick building syndrome”. Childhood asthma has been associated with mould in the home and water damage^36–38^ as a consequence of exposure to fungal volatile compounds, mycotoxins, fungal allergens and cell wall components^39^. Emerging data indicates that acquisition of antifungal-resistant mycoses by individuals can also occur as a consequence of exposures from urban environments in moulds such as *A. fumigatus* where composting processes, both at home and industrially, are known to amplify azole-resistant genotypes^40^. The risk of exposure to fAMR moulds in high risk settings such as hospitals is currently unclear however deserves closer attention; azole-resistant genotypes of *A. fumigatus* have been detected in hospital air and could be acquired by patients during episodes of immunosuppression^41^. More widely, nosocomial outbreaks of multidrug resistant *C. auris* have been repeatedly linked to environmental reservoirs within healthcare settings where the organism is highly persistent due to heightened stress tolerance^30,42^. The high thermal optimum of *C. auris* alongside its adaptation in the face of antifungal drug exposure have led to hypotheses that global warming and widespread antifungal selection are synergizing to open previously inhospitable niches to this pathogen^43^. For this reason, better understanding of environmental co-factors that underpin these taxonomically diverse fAMR pandemic scenarios is urgently required.

### Emerging fAMR and dual-use of antifungals in the environment and clinic

Following early observations of patients acquiring azole-resistant aspergillosis prior to treatment^44^, links between the use of environmental fungicides that share the same molecular target as their clinical analogues (‘dual-use’) and fAMR have been demonstrated. Emerging multidrug (MDR) and multi-mode-of-action (MOA) resistance in the environmental opportunistic mould *A. fumigatus* is a significant clinical concern. With over 20 million people affected annually and global estimates of crude annual mortality exceeding two million, aspergillosis kills more people than any other fungal disease^3^. Because person-to-person transmission is rare to non-existent, azole resistance seen in patients must either have evolved in situ (e.g.^45^ or have been acquired as a consequence of exposure to agricultural DMI’s^46^. The evidence base demonstrating the acquisition of azole-resistant *A. fumigatus* by patients is synoptic and has been recently reviewed^4^. Direct evidence includes: (i) the presence of azole-resistant *A. fumigatus* in environments that are enriched in substrates where adaptation to fungicides is likely^40,47^; (ii) landscape-scale bioaerosolisation of azole-resistant *A. fumigatus* spores^48^; (iiia) a similar genetic identity of CYP51A azole-resistance alleles in the environment and the patient^41,49^ and; (iiib) a similar genomic identity of azole-resistant *A. fumigatus* in the environment, air and patient^50^. Indirect evidence includes: (iv) increases in incidence of clinical azole-resistant aspergillosis expected under a model of continued directional selection by fungicide use^51^ and; (v) the co-occurrence of resistance encoded in the *A. fumigatus* genome to other mode-of-action (MOA) agricultural fungicides that include methyl benzimidazole carbamate, quinone outside inhibitors and succinate dehydrogenase (SDH) inhibitors^52,53^.

The importance of this evidence base should not be underestimated. From a clinical perspective, the occurrence of fAMR in aspergillosis results in a measurable increase in the already significant mortality^54^ and the recent demonstration of widespread resistant spore bioaerosols^48^ speaks to an underlying exposure that is almost unavoidable for those patients with risk factors for aspergillosis. In 2002, the clinical risk of environmentally driven resistance in human fungal pathogens was considered by the EU Health and Protection Directorate-General Commission, who commissioned a committee to provide an ‘Opinion on azole antimyotic resistance’. The conclusion of that scientific steering committee was that they “…*do not consider that the increased resistance to treatment of fungal infections with azole antimycotics is related to the use of azole fungicides in agriculture*”^55^. It is clear that this conclusion must now be rejected, that the emerging spectre of fAMR spans a far greater swathe of the fungal kingdom than just *A. fumigatus*, and that we are perennially underestimating the scale and true extent of the risk. Moreover, it is also clear that this risk has to be addressed within a one health perspective, because there are complex interactions, links and tradeoffs nested within our global use of antifungal chemicals in farming, industry and healthcare.

**Fig. 1:**
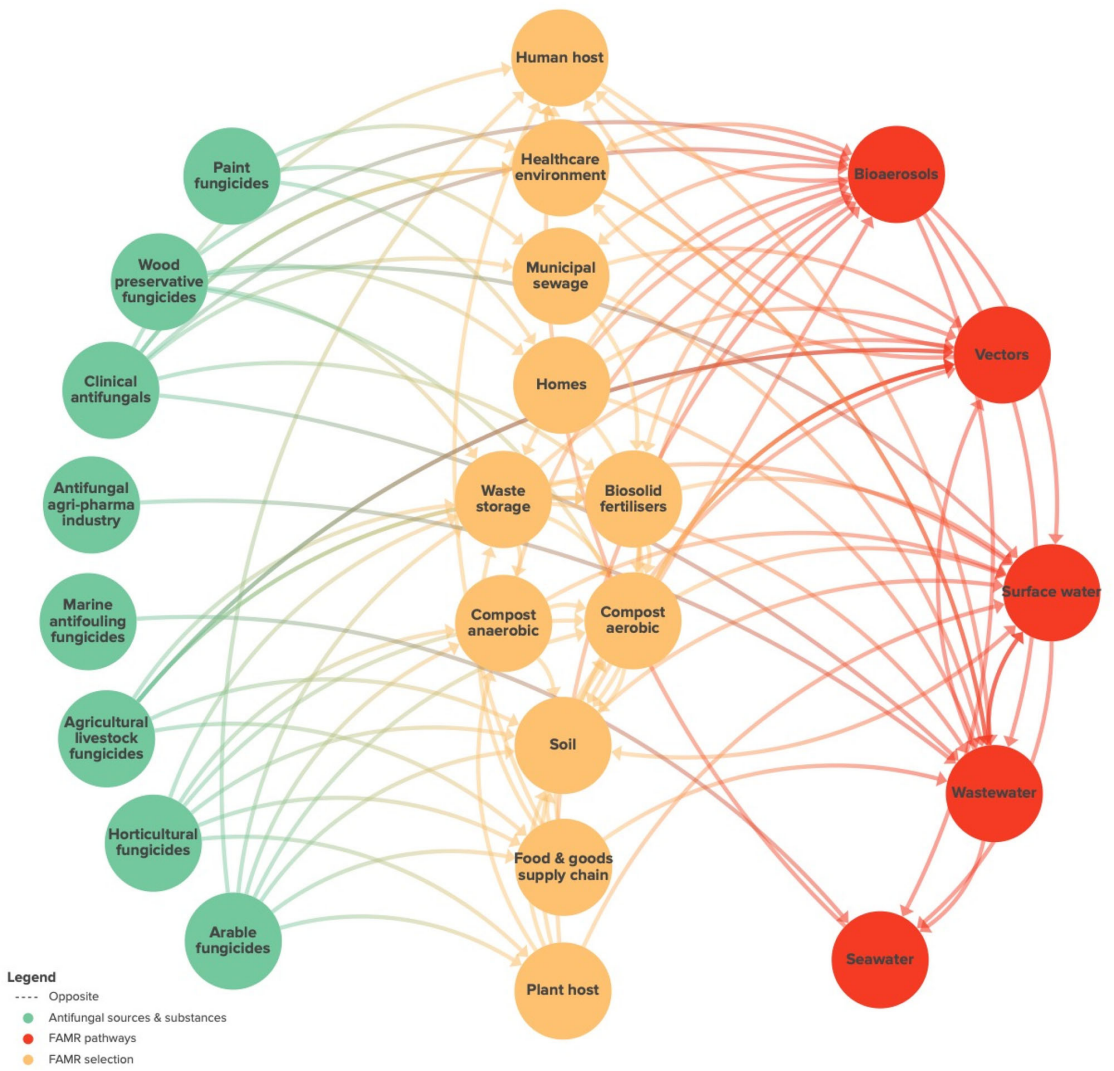
Potential hotspots of fAMR. A generic systems map scoping the potential for sources of antifungal chemicals (green) to interact with ecological compartments where selection and adaptation of fungi to antifungals can occur (orange) that then lead to pathways of dispersal of viable fAMR inocula (red). The figure is generated from a kumu project where it can be dynamically browsed at https://kumu.io/mcfisher/famr-antifungal-sources-pathways-and-vectors.

To examine these interactions more deeply, our group of authors undertook a generic opinion-based systems mapping exercise to conceptualise and visually represent the main putative sources of antifungal chemicals, the contexts where these chemicals could theoretically contact and impose selection on fungi, and the environmental compartments where spread of antifungal resistant inocula could occur (Fig. 1). Clearly, this browsable network map indicates a high level of dimensionality where antifungal chemicals from varied sources can impose selection on a broad range of potentially pathogenic fungi, and that then leads to potential dispersal via water and air. Importantly, this means that any attempt to minimize morbidity and mortality due to fungal AMR involves of a variety of sources (e.g. biomedical, agricultural, industrial), potential reservoirs and hotspots (e.g. recycling, healthcare, agriculture) and routes of dispersal (e.g. waters, vectors and the atmosphere) in order to propose holistic interventions. How to achieve these interventions is not clear given the interlinks between anthropogenic systems leading to potentially large tradeoffs associated with mitigations. To bring clarity to this complex multi-sectorial system, we describe a roadmap towards a more refined one health understanding of fAMR.

### A roadmap for one health understanding of fAMR

Food security is increasingly challenged by climate change, expansion of fungal pathogens and increased fungicide usage^56^. We need to identify practices driving dual-use fAMR, understand hotspots where natural selection occurs and fAMR is amplified, and the risk that resistance poses to food security and the durability of our limited antifungal arsenal in the clinic and environment. This understanding will enable evidence-based interventions to be suggested for safe antifungal use in agriculture, healthcare and other environments, and to avoid/minimise obvious trade-offs.

Here, we advocate for four inter-related focus areas that will enhance our ability to address the spread, and mitigate the risk, of fAMR:

1. Integrating antifungal development, risk assessment and policy across one health
2. Upgrading surveillance of antifungal resistance across one health
3. Defining hotspots in the generation of fungal antimicrobial resistance
4. Determining the social and economic drivers of antifungal deployment, fAMR reservoirs, and interventions

### Integrating antifungal development, risk assessment and policy across one health

Better understanding of the underlying mechanisms underpinning fAMR that includes both existing classes of fungicides, new environmentally durable fluorinated forms and novel drug MOA classes is required. We need deeper insights into the impact of fungicides on off-target fungal communities that may lead to disruption of ecosystem services or release of pathogenic fungi from competitive constraints. These insights will inform the development and refinement of more relevant risk-assessments and mitigation strategies that need to be deployed to reduce the evolution of resistance as well as the emergence of novel antifungal-resistant human fungal pathogens (see also Area 4). The growing prominence of clinical azole resistance has prompted significant investment in a new generation of novel antifungal drug classes. Olorofim (DHODH inhibitor) and fosmanogepix (Gwt1 inhibitor) are expected to be licensed clinically within 2 years^57^. Simultaneously, analogous compounds have been developed that have, or are close to receiving, approval for use in agriculture, including the DHODH inhibitors ipflufenoquin and quinofumelin^58^ and the Gwt1 inhibitor aminopyrifen^59^. There is rapidly growing concern that the widespread use of agents with the same MOAs in agriculture and medicine will once again drive resistance in the environment and onwards to the clinic in both opportunistic pathogenic filamentous fungi and yeasts. It has recently been shown, as an example, that ipflufenoquin can select cross resistance to olorofim in *A. fumigatus* without impacting in vitro fitness^60^.

### Key priority areas

**Fig. 2:**
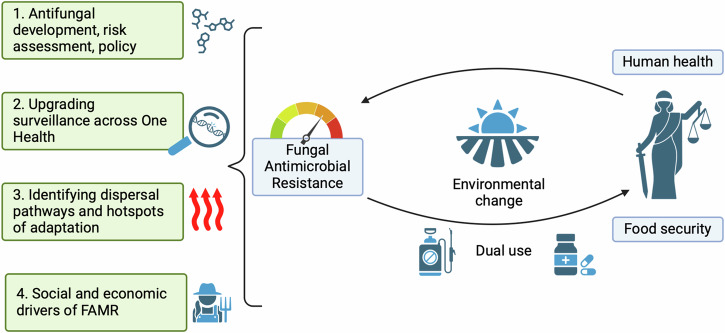
Key priority areas to better understand fAMR. Priority areas where evidence and integration is needed to understand the ecological and evolutionary drivers of emerging fungal antimicrobial resistance. ‘Lady Justice’ represents the trade-offs inherent in the dual-use of antifungals with similar modes-of-action spanning clinical and environmental settings. Created under licence in BioRender. Fisher, M. (2024) BioRender.com/y84s888.

There is an urgent need to understand the temporal, and spatial scale of fAMR. This will require the development of experimental ecological methods to understand the potential for resistance selection and amplification in relevant ecological settings, and to use these insights to modernise the risk-assessment of fungicide and antifungal chemicals. Within the context of environmental fAMR hot-spots in waste streams (Fig. 1), we do not have a clear definition of the fungicide concentrations that are safe with respect to selection of resistant, tolerant or persistent isolates. Subsequently, we need a better understanding of how long fungicides persist and disperse in the environment and how this relates to their stability and capacity to drive resistance. For *A. fumigatus*, amplification of pre-existing resistance alleles is the primary mode by which resistance has been shown to emerge and spread. However, we need to understand and develop risk models based on understanding the fitness consequences of resistance alleles in order to predict the likelihood of resistance persisting in the absence of selection. We need to better understand the efficacy of fungicide combinations or multi-mode-of-action class fungicides targeting more than one molecular target, and to examine best-case usage scenarios for antifungal chemicals that take into account one-health aspects of fAMR. There is a need to understand the full environmental and societal case for the use of fungicide products and their combinations, and to assess why agro-industry persists in the use of single-site fungicides. Within this context, development of predictive mathematical and economic models to enable forecasting of one health dual use fAMR risk would be invaluable Fig. 2.

### Upgrading surveillance of antifungal resistance across one health

There is currently limited fungal genomics capacity for global molecular surveillance of fAMR, although international academic efforts to implement fungal genomic surveillance of both MDR *C. auris* and azole-resistant *A. fumigatus* have begun^42,50^. Clinical implementation requires validation of utility, feasibility and cost effectiveness for species identification, outbreak analysis, prediction of resistance, and development of standard operating procedures, bioinformatic pipelines and laboratory implementation. Genomic data requires integration with rapid molecular tests, phenotypic assays and drug susceptibility testing, all of which need to be enhanced with regard to performance for detecting/identifying resistance across both clinical and natural environments. These efforts require coordination with environmental monitoring approaches across one health, for example surveillance of airborne spores in agriculture, and healthcare, with effective communication across research communities.

In the United Kingdom, the Government’s PATH-SAFE program (Pathogen Surveillance in Agriculture, Food and the Environment), led by the Food Standards Agency, is developing a national surveillance system for foodborne disease and bacterial AMR and has been researching the potential impact of antifungals that may drive selection in soil and water. PATH-SAFE tools have recently been adapted to assess *C. auris* inter-hospital transmission dynamics (https://pathogen.watch) and the program aims to create national data system for pathogen sequence and metadata, new surveillance approaches and genomic diagnostic methods ‘in field’. The WHO has launched AMR surveillance of invasive fungal infection as part of the Global Antimicrobial Resistance Surveillance System[GLASS^61^] and there are wider efforts within the European Union and the USA CDC to expand and link fAMR surveillance that testify to a trend in wider surveillance of fAMR.

### Key priority areas

There is an urgent requirement for a wider and more transnationally-integrated fungal surveillance framework across the diversity of areas covered by the one health aspects of fAMR. This will allow for investigation of spatial and temporal trends in the molecular epidemiology of resistance in humans, animals, plants and the environment. This framework should be integrated with current international surveillance actions and funding (e.g. WHO GLASS surveillance system, CDC Emerging Infections Program, JPIAMR INFORM-AFR) alongside strengthening local environmental quality monitoring if it is to be maximally useful. UK PATHSAFE has provided a strategy towards surveillance of AMR in airborne fungal pathogens that could use existing field sampling equipment and protocols.

We need to develop common technical approaches, surveillance techniques and standards for multiple fungal pathogens, underpinned by enhanced and standardised detection and diagnostic methods that can be used across a range of contexts and expertise. Key requirements include development and validation of integrated genomic protocols, and standardised bioinformatic pipelines for outbreak analysis, phylogenetic studies and identification of antifungal resistance. To complement next generation sequencing approaches, standardised approaches for rapid and accurate field/near-patient tools to identify fAMR (easy to use phenotypic susceptibility methods, novel and equitable diagnostic assays, point of care genome sequencing technology such as Nanopore DNA sequencing) across one health should be adopted. Additionally, assessment of emerging technologies such as metagenomics to identify microbial communities predictive of fAMR are needed.

These approaches will empower a broader adoption of rapid field/near-patient testing and identification of environmental and biological reservoirs and spread. Access for lower resource settings needs to be prioritized and suitable economic models adopted. To further bolster microbiologically-driven surveillance systems, development of clinical data pipelines across scales in partnership with healthcare providers and citizen science approaches is needed. These should complement and integrate with better standardisation of surveillance of agriculture, the environment, healthcare facilities and atmosphere by adopting shared methods. These systems need to be able to define modes of transmission, for instance *via* air for moulds, or by direct patient-to-patient contact for dermatophytes or yeasts. The effect of global trade on movement of plant pathogens also needs to be better understood. To further enable underpinning research, we need to establish fungal biorepositories and metadata to accelerate academic-industry collaboration, with a model example being the existing UK CF Trust AMR Syndicate (https://cfamr.org.uk). Currently, all surveillance schemes are voluntary and this will likely bias as well as limit outputs.

### Defining hotspots in generating fungal antimicrobial resistance

Antifungals are widely used across human/veterinary medicine, in personal care products, and as pesticides in agriculture and horticulture; they may concentrate in the environment through agricultural run-off and composting, hospital wastewater, domestic sewage and biosolid amended soils [^62^; Fig. 1]. In the EU, fungicides account for 40% of total pesticide sales, which is predicted to increase due to climate change, emerging resistance and invasive fungal species^62^. Europe alone produced and disposed of 8.97 million tons of sewage sludge in 2020 and across the EU, approximately 50% is used as a fertiliser^63^. Additionally, climate change means use of wastewater for irrigation is increasingly important^64^. UK Government regulations for use of sewage sludge as a fertiliser primarily focus on permissible concentrations of potentially toxic elements (PTEs), helminths and *Salmonella*; fungicides and fungi are currently ignored^65^. A similar situation occurs in the European Union (Council Directive 86/278/EEC). Whilst there is a growing awareness of the prevalence of undegraded antibiotics and antimicrobial resistance genes in sewage sludge and soil following deployment^66^, this is currently lacking for fAMR^67^. This is particularly important for antifungals such as fluconazole, which are often excreted unchanged in urine and can therefore accumulate in wastewater.

There are concomitant potential threats to human health via the food-chain and from processing sewage sludge and wastewater (e.g. bioaerosolisation of fAMR) and this is extended to animal husbandry (e.g. azole resistant *A. fumigatus* present in poultry litter, which can be used as cattle feed)^68^. Waste concentration of antifungals leading to amplification of resistance is a key area of concern. In the EU and UK, approximately 1.4 billion tonnes of manure is produced annually; this is predicted to rise, driven by a 17.8% projected increase in poultry production by 2030 (OECD/FAO 2021). In the UK, quality of composts and anaerobic digestates is encouraged through certification schemes such as PAS100 and PAS110 respectively; these standards specify limits for coliforms and potentially toxic elements, but do not include fungicide indices. Furthermore, current UK Environmental Risk Assessment guidelines do not require risk assessment for fAMR and there are no standardized protocols to generate such data despite the evidence that composts are enriched for azole-resistant *A. fumigatus*^40^. Consequently, there is minimal insight into the selective potential of antifungals to induce fungal resistance at environmentally-relevant concentrations.

Whilst fungicide concentrations in river catchments may be temporal, the consistency of detection of multiple fungicides (including azoles) resulting from agricultural/horticultural runoff is concerning, particularly as there is a paucity of information on the effects on aquatic fungi, or of the combined effects of multiple drugs^69^. Furthermore, wastewater treatment plants are a key source of clinical and personal care fungicides entering rivers, and persistence in watercourses is likely^70,71^. This is a global concern, particularly where sanitation is limited, as exemplified by Monapathi et al. who demonstrated widespread fungal resistance of yeasts (including opportunistic pathogens) isolated from two rivers in South Africa^72^. Aquatic biota are therefore chronically exposed to low or moderate fungicide concentrations, but much higher concentrations can accumulate during storms, in agri-intensive parts of the year and in regions with intense fungicide use^62^. Furthermore, climate change-related fungal adaptation to heat stress may increase the likelihood of acquiring fungal diseases from contaminated waters^73^. *C. auris*^74^, *C. albicans*^75^ and *A. fumigatus* are found in aquatic environments, but the impact of aquatic fungicide exposure on fAMR is less well understood^62^. Recent progress for mass spectral data-mining of antifungal agents in wastewater has been made to inform epidemiological analyses^71^. Better definitions are required on the contribution of waste streams and recycling to fAMR, so that approaches can be developed to mitigate their influence. Systematic risk assessment and analysis of drivers of waste-generating practices and technologies in close cooperation with academics, regulators and end-users will enable better understanding of likely contributory factors.

### Key priority areas

Science, users and actors in policy need to define the impact of evolving technologies around production and application of organic amendments to soils including biosolids (sewage and farm derived) and green waste composts on the induction and selection of fAMR. A standardised framework to evaluate the importance of organic soil amendments as ‘hotspots’ of fAMR and the risks of subsequent transfer to other environmental compartments through agricultural practices (e.g. soil, surface & ground water and atmosphere via dust and bioaerosols) is needed.

Since fungicide residues and azole resistant *Candida* sp. have been reported in surface and groundwaters^75^, evaluation of the impact of soil organic amendments, runoff from soils, and wastewater inputs into rivers as drivers of fAMR in surface- and groundwater is required. fAMR pathways into, from (e.g. water abstraction, irrigation), and within, rivers should also be assessed. Land use needs to be incorporated into risk assessments for the promotion of fAMR from urban and agricultural land. With increasing urbanisation, run-off from gardens, household wood preservatives and pharmaceuticals may be a significant source of fAMR into surface waters and also requires further evaluation.

The safe thresholds for ‘environmental’ concentrations of azoles or other antifungals in waste streams and surface waters, and the minimal selective concentration for the antifungals present need to be defined. Low azole concentrations may also promote fAMR in biosolids, manures, composts and soil, and azole degradation kinetics within wastewater treatment plants needs to be characterised to be able to calculate the consequences of intermittent contamination of environmental ecosystems. Better understanding of these factors will allow mitigation frameworks to be established or improved to address; for example, limiting run-off from agricultural fields via buffer strips or bioremediation of affected riparian zones between fields and water courses, or by creating novel barriers of buried biochar to capture run-off and promote fungicide biodegradation.

### Determining the social and economic drivers of antifungal deployment, fAMR reservoirs, and interventions

The strategic use of fungicides in agriculture is part of integrated pest management (IPM), which encompass good practices such as crop rotation, the use of resistant crop varieties, plant disease monitoring and forecasting, and various methods of physical, biological and chemical control. Anti-resistance stewardship measures are regarded as integral to IPM, and are based largely on the principle of reducing reliance on any one individual class of fungicides. As such reduced doses, mixtures and alternations are promoted where possible within the framework of IPM. Implementing measures, although beneficial for the industry as a whole, can carry cost to the individual. Hence stewardship uptake can be sub-optimal, so statutory label requirements to reduce the risk of resistance are often imposed by regulators as a condition for approval and use. Fungicides are assessed for resistance risk prior to launch and stronger mitigation measures identified for those deemed at high risk^76^. In the UK, the Fungicide Resistance Action Group (FRAG;^77^) gathers and interprets information on fungicide resistance and its management. The group convenes consensus views for the UK, including stewardship guidance for users and advice to the pesticide regulatory authority in the UK. At an international level, the Fungicide Research Action Committee (FRAC, https://www.frac.info) represents agrochemical industries and works to 1) prolong the effectiveness of fungicides liable to encounter resistance problems and 2) to limit crop losses should resistance occur. Similar independent fungicide resistance action groups exist in other countries e.g. the Baltics (Nordic Baltic Collaboration About Pesticide Resistance NORBARAG) and Australia (Fungicide Resistance Extension Network AFREN).

However, knowledge of the resistance status of crop pathogens is often incomplete or slow to emerge. Monitoring and surveillance programmes are largely funded by the agrochemical industry with more limited research in the public domain. Minor pathogens of smaller crops receive little attention. National surveys on the health status of crops are also incomplete, and for example those conducted in the UK by the Defra (the UK Department for the Environment, Farming and Rural Affairs) and by Scottish Government are confined to major combinable crops and only record the diseases found, not their resistance status.

PATH-SAFE is developing approaches to measure antimicrobials in biosolids and surface waters. ESPAUR (the English Surveillance Program for Antimicrobial Usage and Resistance) produces top level data for antifungals, although the UKHSA “Fingertips” resource for antibiotic use and resistance does not currently extend to antifungals. Even less is known about antifungal usage across the built environment and in companion animals, and the recent detection of *C. auris* in both feral and companion-dogs in the USA speaks to the risk of spillover into other vertebrates that may act as vectors, reservoirs or amplifiers of fAMR^78,79^. Currently, international focus is patchy, but there are signs of increasing transnational integration with international funding (JPIAMR) and WHO (GLASS) to better understand reservoirs and vectors of fAMR.

For clinical antifungal stewardship, an evidence base is lacking compared to antibacterials^80^. Optimal stewardship strategies are complex due to the heterogeneity of patient cohorts, fungal pathogens, and personalised diagnostic strategies and treatments^80^. Key principles for antifungal stewardship include optimisation of prescribing through better diagnostics, appropriate antifungal susceptibility testing to ensure correct treatment regimens are given, antifungal therapeutic drug monitoring and appropriate de-escalation of therapies. Stewardship is delivered through highly specialised multidisciplinary teams and was reinforced in the UK by the National Health Service (NHS) national Antifungal Stewardship Implementation Pack in 2019^81^. However, it is more problematic to assess the scale of over-the-counter sales of fungicides, such as topical antifungals aimed at controlling skin/nail or genital fungal infections.

Establishing the most cost-effective chemical and non-chemical interventions to mitigate the emergence of antifungal resistance requires agricultural and health economic modelling. Establishing feasibility of implementation in different contexts requires social research and pragmatic piloting with affordable monitoring and evaluation; this is especially relevant for resource poor settings where the evidence-base for stewardship is low to non-existent.

### Key priority areas

We need to develop structured approaches to measure antifungal deployment with a one health perspective, and an integrated stewardship intervention framework to limit fAMR. A one health approach is needed that enables judicious use of antifungals across agricultural, veterinary and human health settings to mitigate the spread of fAMR while accepting the pressures each area is working within.

To achieve this, integrated data pipelines on antifungal deployment across healthcare and environmental settings need to be established. Together with enhanced surveillance on fungal disease prevalence and fAMR, this will guide targeting of local, regional and national antifungal stewardship interventions. A mix of interventions is likely to be required to enable local antifungal stewardship quality and innovation programs to be implemented to improve patient care; these interventions will require integrated health economic models and clinical assessment tools to drive optimal prescription of antifungals that are relevant across different socio-economic and global settings. Subscription-based purchasing models have been recently introduced for antibacterials by NHS England. This enables stewardship of high-value agents whilst incentivizing pharmaceutical companies to develop novel agents. Extension of subscription-based models for antifungals, based on Health Technology Assessment of clinical and cost effectiveness with relevant pharmaceutical companies and health boards needs to be considered.

Within agriculture, refinement of cropping systems (e.g. crop diversification or rotation) and development of economic evaluation frameworks to enable farmers to implement antifungal stewardship and manage resistance, is needed. These illuminate the trade-offs involved in changing fungicide use and integrating alternative control methods (e.g. integrated pest management). Encouraging greater use of disease resistant crop varieties, including those developed by advanced precision breeding technologies as well as genetic modification, offers a durable alternative to chemical control of fungal diseases. Advances in our understanding of plant immunity now offer multiple routes to achieving durable disease resistance^82^, including the use of multiple disease specificities in cereals^83^. The impact of stewardship measures also need to be modelled in terms of implementation costs, production effects and effectiveness in reducing antifungal resistance, especially if used in tandem with genetic means of disease control. Better understanding of farmers’ behaviour towards fungicide use, and how it is affected by risk perception, knowledge and other technical and economic factors, is essential when scaling-up intervention measures. Increased understanding of the role of applying materials to land can play in the further deposition of antifungal agents on agriculture is also needed—for instance by replacing synthetic nitrogen-based fertilisers with on-farm windrow composters. Evaluating the key drivers of increasing fungicide usage globally and how this relates to climate change is also required.

In addition to documenting potential social pressures and solutions that can impact fungicide use, social research is needed to help to understand drivers of both fungal infection and antifungal exposure that might be amenable to change. In the context of increasing antifungal resistance, and the potential for the burden of infection to fall unevenly on those most vulnerable to infection and least able to fund next-line therapies, identifying social, economic and regulatory changes that can reduce this burden will become critical.

## Discussion

‘Environmental change’ in terms of the disturbance in both abiotic (physical) and biotic (biological) factors, has been accelerated by human activity. Given the extent that the fungal kingdom is interwoven into the ecology of the habitable planet, it is no surprise then that environmental change is reflected by alterations in fungal distributions and traits, which then translates to changes in mycoses across space and time. Dual use of antifungals has led to the emergence of fAMR across plant and human fungal pathogens, threatening food security, animal and human health. In the context of the limited arsenal of antifungals available for clinical use, this has been driven primarily by widespread usage of azoles fungicides in agriculture. However, there are significant concerns that the next generation of antifungals in clinical development are also now threatened through dual use as agricultural fungicides with the same MOA. These concerns are set against a background of trade-offs between the competing imperatives of food security, agricultural and health economics, fungal ecosystem services and human health. To address these multi-sectorial issues, innovative one health approaches are urgently needed bringing together transdisciplinary researchers, as well as key stakeholders across agriculture, health and end-users. Key priorities (Table 2) to enable the mitigation of fAMR include experimental approaches to define the underlying mechanisms of fAMR emergence, integrated fAMR surveillance across one health, systematic assessments of waste streams and their roles in generation of fAMR, and better understanding of the social and economic factors that need to be considered when developing novel antifungal stewardship interventions.

**Table 2:** Key recommendations to better manage the emergence and spread of fungal antimicrobial resistance across Priority Areas 1–4

fAMR recommendation		
1. Integrating antifungal development, risk assessment and policy across one health	1-1	Develop experimental ecological methods to understand the potential for resistance selection and amplification in relevant settings; use these insights to modernize the risk-assessment of fungicide and antifungal chemicals
	1-2	Monitor shifts in the type and use of antifungal chemicals in agriculture / human healthcare to better understand future selection pressures and to develop best-case-scenarios
2. Upgrading surveillance of antifungal resistance across one health	2-1	Build a wider and more transnationally integrated fungal surveillance framework across the diversity of areas covered by one health aspects of fAMR
	2-2	Foster common technical approaches, surveillance techniques and standards for multiple fungal pathogens, underpinned by enhanced standardized detection / diagnostic methods and genomic epidemiology
3. Defining hotspots in generating fungal antimicrobial resistance	3-1	Develop a standardized framework to evaluate the importance of land use practices and the risks of developing hotspots with subsequent impact on other environmental compartments
	3-2	Establish safe thresholds for environmental concentrations of antifungals in waste streams; define the minimal selective concentration for the antifungals
4. Determining the social and economic drivers of antifungal deployment, fAMR reservoirs, and interventions	4-1	Develop structured approaches to measure antifungal deployment, and an integrated stewardship intervention framework to limit fAMR. A one health approach is needed that enables judicious use of antifungals across agricultural, veterinary and human health settings
	4-2	Within agriculture, refinement of cropping systems and development of economic evaluation frameworks to enable farmers to implement antifungal stewardship and resistance management
